# Evolving Therapeutic Landscape of Intracerebral Hemorrhage: Emerging Cutting-Edge Advancements in Surgical Robots, Regenerative Medicine, and Neurorehabilitation Techniques

**DOI:** 10.1007/s12975-024-01244-x

**Published:** 2024-04-01

**Authors:** Danyang Chen, Zhixian Zhao, Shenglun Zhang, Shiling Chen, Xuan Wu, Jian Shi, Na Liu, Chao Pan, Yingxin Tang, Cai Meng, Xingwei Zhao, Bo Tao, Wenjie Liu, Diansheng Chen, Han Ding, Ping Zhang, Zhouping Tang

**Affiliations:** 1 Department of Neurology, Tongji Hospital, Tongji Medical College, Huazhong University of Science and Technology, Wuhan, Hubei China; 2 School of Mechanical Science and Engineering, Huazhong University of Science and Technology, Wuhan, Hubei China; 3 School of Astronautics, Beihang University, Beijing, China; 4 Beijing WanTeFu Medical Instrument Co., Ltd., Beijing, China; 5 Institute of Robotics, School of Mechanical Engineering and Automation, Beihang University, Beijing, China

**Keywords:** Brain-computer interfaces, Intracerebral hemorrhage, Minimally invasive surgery, Neuronal reprogramming, Stem cell transplantation, Surgical robots

## Abstract

Intracerebral hemorrhage (ICH) is the most serious form of stroke and has limited available therapeutic options. As knowledge on ICH rapidly develops, cutting-edge techniques in the fields of surgical robots, regenerative medicine, and neurorehabilitation may revolutionize ICH treatment. However, these new advances still must be translated into clinical practice. In this review, we examined several emerging therapeutic strategies and their major challenges in managing ICH, with a particular focus on innovative therapies involving robot-assisted minimally invasive surgery, stem cell transplantation, in situ neuronal reprogramming, and brain-computer interfaces. Despite the limited expansion of the drug armamentarium for ICH over the past few decades, the judicious selection of more efficacious therapeutic modalities and the exploration of multimodal combination therapies represent opportunities to improve patient prognoses after ICH.

**Supplementary Information:**

The online version contains supplementary material available at 10.1007/s12975-024-01244-x.

## Introduction

Intracerebral hemorrhage (ICH) is widely recognized as the most devastating and refractory subtype of stroke, characterized by a high fatality rate of approximately 40% and dismal functional outcomes, with only 25% of patients attaining functional independence 6 months after onset [1, 2]. The third Intensive Care Bundle with Blood Pressure Reduction in Acute Cerebral Hemorrhage Trial (INTERACT3), a groundbreaking study, has recently shown that early intensive blood pressure reduction, strict control of blood glucose levels, temperature management, and prompt correction of abnormal anticoagulation can significantly improve functional outcomes in ICH patients, providing level I evidence that a comprehensive care and treatment paradigm can significantly optimize functional outcomes in patients with ICH for the first time [3]. Despite substantial progress, there remains a notable paucity of proven interventions aimed at improving clinical outcomes when considering the significant impact this disease has on patients’ lives. As such, it is very important to allocate resources to research and development to identify novel and effective therapeutic approaches that can address this pressing issue.

**Fig. 1:**
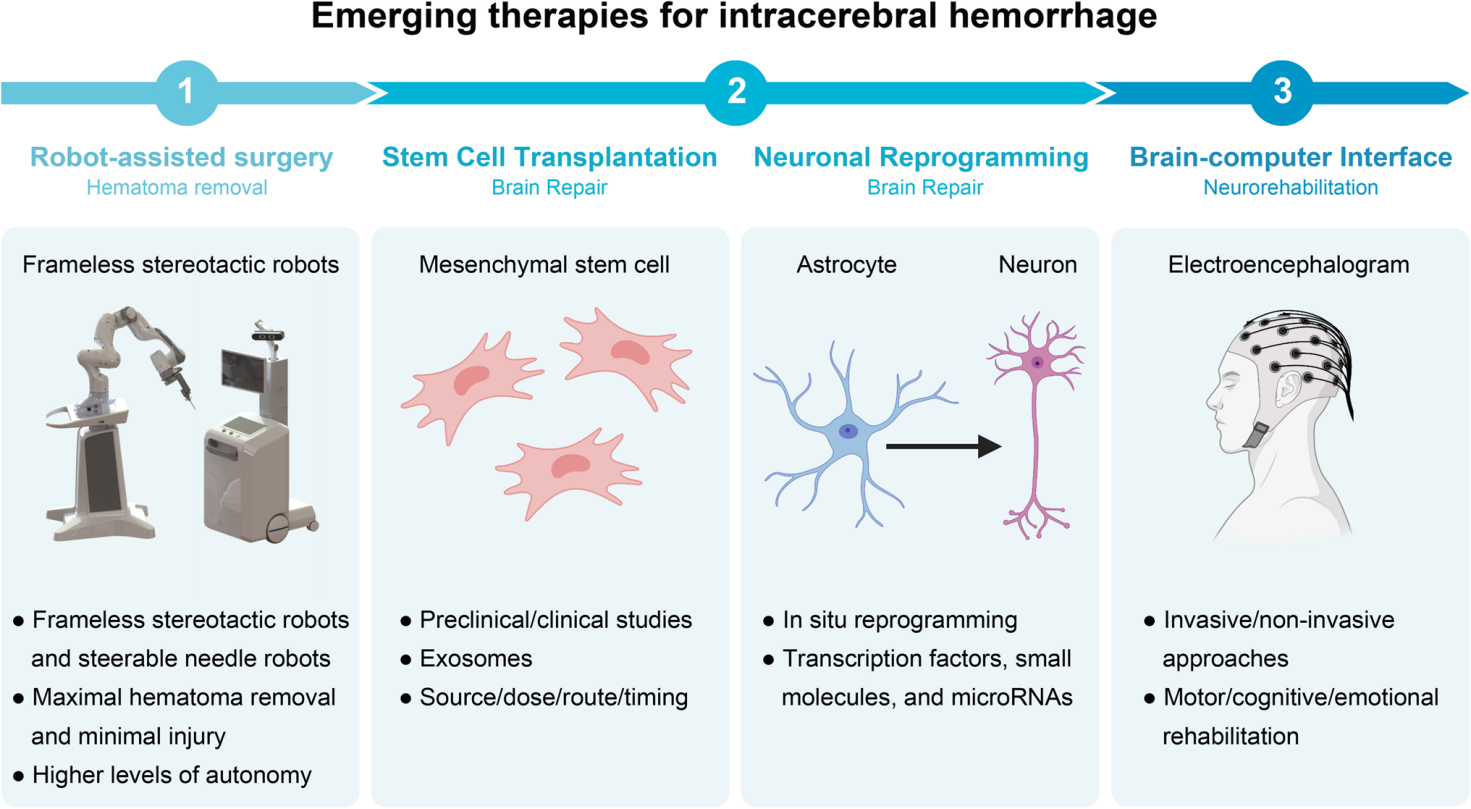
Emerging therapies for intracerebral hemorrhage

In fact, the majority of adverse outcomes following ICH can be attributed to the primary brain injury caused by the hematoma itself and secondary brain injuries resulting from pathophysiological responses (e.g., brain edema, neuroinflammation, oxidative stress) to hematoma metabolites [4]. Therefore, in theory, to effectively treat patients with ICH, the following steps are necessary. First, for primary brain injury, timely removal of the hematoma, hemostasis, and prevention of rebleeding is required to alleviate the pressure on the brain tissue and the production of toxic metabolites. Second, to prevent secondary brain injury, measures such as reducing brain edema and suppressing neuroinflammation should be implemented. Finally, it is essential to promote neuroregeneration and rebuild neural circuits in response to neuronal loss and resulting neurologic impairment. Recent advances in the fields of robot-assisted surgery, regenerative medicine, and neurorehabilitation have opened up the possibility for improving the prognosis of ICH. Specifically, this review describes four cutting-edge techniques for the management of ICH that focus on addressing these three essential aspects of effective treatments (Fig. 1): (i) robot-assisted minimally invasive surgery (MIS); (ii) stem cell transplantation; (iii) in situ neuronal reprogramming; and (iv) brain-computer interfaces (BCIs).

## Search Methods and Study Eligibility

**Fig. 2:**
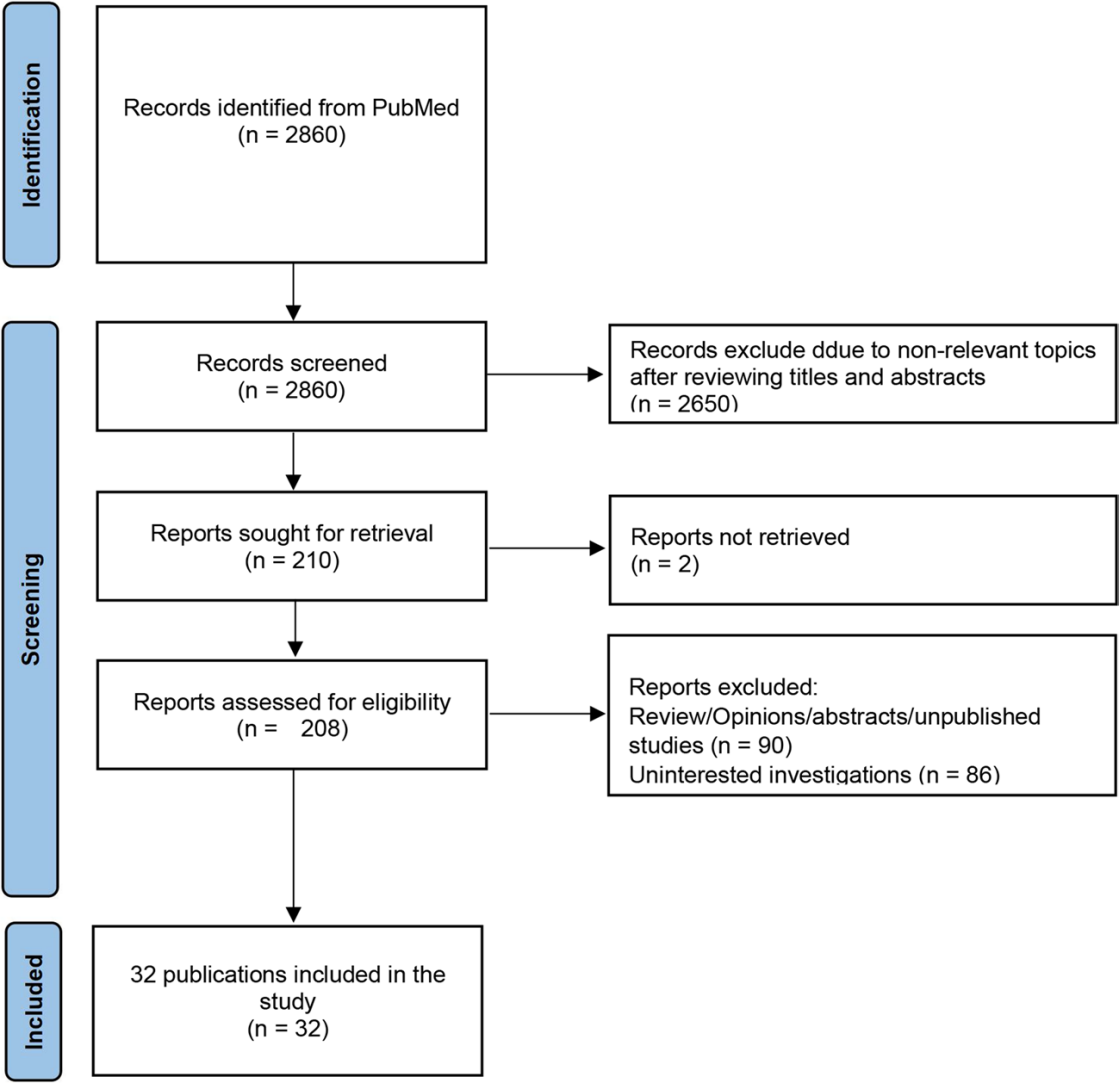
Flow diagram of study identification and selection

A systematic search was conducted in PubMed for all articles published from database inception to March 1, 2024. The Preferred Reporting Items for Systematic Reviews and Meta-analyses (PRISMA) 2020 reporting guideline was leveraged to guide the study [5]. The search terms used including “intracerebral hemorrhage,” “cerebral hemorrhage,” “brain hemorrhage,” “hemorrhagic stroke,” “hematoma removal,” “hematoma evacuation,” “robot,” “mesenchymal stem cell,” “neuronal reprogramming,” “brain-computer interface,” and “brain-machine interface.” In order to identify any additional relevant articles, a manual reference search of the included articles was conducted. Exclusions were made for reviews, opinions, abstracts, and unpublished studies. In cases where manuscripts were unavailable, authors were contacted to obtain copies if necessary. Two independent reviewers (DYC and ZXZ) conducted a screening of search results based on titles and abstracts, with any conflicts resolved by involving a third reviewer (PZ). The search results are presented in Fig. 2.

## Robot-Assisted MIS for Hematoma Removal

Craniotomy and MIS are the two primary surgical approaches utilized for hematoma evacuation [6]. Craniotomy imposes more rigorous demands on both equipment and personnel training, and it is possible that the inevitable impairment of normal brain tissue during surgical maneuvers nullifies some of the benefits of the hematoma evacuation [7]. The International Surgical Trial in Intracerebral Hemorrhage (STICH) and STICH II have conclusively established that conventional craniotomy is ineffective in enhancing the prognosis of patients with ICH and in augmenting their survival rate when compared to initial conservative treatment [8, 9]. Hence, MIS appears to be the most promising surgical approach for hematoma evacuation; MISs include neuroendoscopic surgery and stereotactic hematoma puncture and drainage. Notably, stereotactic hematoma puncture and drainage, which can be executed under local anesthesia and offers several additional benefits, such as reduced trauma, uncomplicated surgery, and minimal equipment requirements, has emerged as one of the most prevalent surgical techniques in clinical settings [7, 10]. The findings of the Minimally Invasive Surgery Plus rt-PA for Intracerebral Hemorrhage Evacuation (MISTIE III) trial demonstrated that while MIS reduced all-cause mortality, it did not confer any functional outcome benefits at 1-year follow-up after ICH [11]. However, the findings from the subgroup analysis indicated that patients who attained the surgical objective of residual hematoma volume (≤ 15 mL) had associated improved functional outcomes, with a concomitant 10% increase in the probability of functional independence after 1 year for each 1 mL of hematoma evacuated beyond 15 mL (*P* = 0.002) [11, 12]. Indeed, currently, MIS is performed by manual puncturing, which is prone to catheter deviation, resulting in suboptimal therapeutic efficacy [13]. Furthermore, the lack of real-time data on hematoma volume and intracranial pressure (ICP) fluctuations during surgery, coupled with the fact that the aspiration procedure is entirely reliant on the operator’s expertise and proficiency, poses a significant challenge. To guarantee the safety of the procedure, the aspiration volume is often limited to a moderate level, which may lead to an unsatisfactory residual hematoma volume. Undoubtedly, all of the aforementioned factors can exert a profound influence on the amelioration of postoperative neurological function in patients with ICH. Overall, in the context of MIS for ICH, the ultimate objectives are to minimize damage to healthy brain tissue and to maximize the evacuation of the hematoma.

Compared to general MIS, robot-assisted MIS is notable due to its exceptional safety features and potential for achieving high efficacy in hematoma evacuation. Its high efficiency is attributed to its remarkable localization accuracy, abbreviated operation time, and impressive anti-jamming capability. The advent of robot-assisted surgery heralds a thrilling new epoch in MIS for ICH, which is regarded as a critical direction of future research. At present, MIS robots used for ICH primarily comprise two types: frameless stereotactic robots and steerable needle robots. Frameless stereotactic robots have a diverse array of functionalities including surgical path planning, navigation, and control software, and typically employ robotic arms endowed with multiple degrees of freedom to facilitate accurate positioning of the puncture point [14]. Markerless techniques (e.g., optical camera image processing) are employed to achieve coordinate registration for these robots, which obviates the requirement for a metal stereotactic frame or fiducial markers secured on the patient’s head; this is how the term “frameless stereotactic robots” was coined [15]. Frameless stereotactic robots, e.g., ROSA robots [16–18], Remebot robots [19, 20], and CAS-R-2 robots [21], have emerged as the preeminent technology in the realm of robot-assisted surgical treatment of ICH and have been extensively utilized in clinical investigations. Xiong et al. undertook a comprehensive systematic review and meta-analysis to ascertain the safety and effectiveness of these three aforementioned robots for drainage catheter placement within the hematoma in patients afflicted with ICH. The results suggest that robot-assisted MIS is a safe and effective modality that surpasses conventional surgical techniques or conservative management strategies in terms of reducing intracranial infection and rebleeding rates, as well as improving neurological function [22].

However, frameless stereotactic robots utilize a straight, rigid stereotactic sheath to access the hematoma, thus failing to circumvent vital cerebral structures featured along the puncture route, and complete hematoma clearance is not achievable in most cases [23]. Soft robots, defined as robots constructed with soft materials [24], are inherently safe and compliant, making them promising solutions to address challenges based on their unique properties and capabilities. Barnes et al. developed and assessed a new soft robotic system for MIS, enabling precise sub-millimeter positioning of the catheter tip. Nevertheless, due to its 10-mm width, the system is not suitable for hematoma evacuation, given that standard brain catheters typically have diameters of a few millimeters or less [25]. Based on the concept of soft robots, a novel rigid-flexible-soft puncture needle has been proposed by our laboratory, which enables the cannula to adapt to variations in tissue stiffness of the skull, brain tissue, and hematoma, thereby mitigating the potential for iatrogenic injury [26]. Furthermore, advancements in steerable needle robot technology, most notably expanded distal workspaces, present alternative and innovative solutions to this challenge. These robots penetrate the hematoma in a linear trajectory, but upon arrival, a precurved inner tube is deployed from the straight outer tube to extract the hematoma, allowing access to a considerably larger spatial scale within the hematoma [27, 28]. A refinement of the typical needle robot was attempted by Yan et al., who proposed a continuum robot design that features nonlinear insertion and dexterous tip manipulation, resulting in more complete hematoma clearance and minimal tissue trauma [29]. Despite the apparent benefits, the majority of steerable needle robots have yet to showcase their potential beyond the confines of laboratory settings, and their clinical efficacy is yet to be established.

## Regenerative Medicine Strategies for Brain Repair

ICH-induced brain injuries give rise to persistent neuronal loss, which is primarily attributable to the limited regenerative potential of adult mammalian brains. Over the past few decades, regenerative medicine has predominantly centered around two highly promising approaches involving the use of either exogenous or endogenous cell sources to solve this problem [30]. As examples of the two methods, stem cell transplantation and in situ neuronal reprogramming have opened up the possibility of better neuronal replacement and repair in ICH [31].

## Stem Cell Transplantation

Stem cell transplantation is a fascinating frontier area of research on neurological diseases, including ICH. Notably, among the diverse types of stem cells, mesenchymal stem cells (MSCs) have garnered vital attention because of their exceptional properties, including their ability to proliferate extensively and differentiate into various cell types, easy isolation from different tissues, low immunogenicity, paracrine activity, immunomodulatory function, and lesser ethical concerns. To date, MSCs have been successfully extracted from a wide range of tissues, such as bone marrow, adipose tissue, dermis, peripheral blood, synovial fluid, dental pulp, periosteum, skeletal muscle, and various neonatal tissues, including umbilical cord, cord blood, placenta, amniotic fluid, and amniotic membrane, and subsequently characterized [32, 33].

The originally anticipated mechanism of stem cell treatment was straightforward: stem cells were purported to supplant impaired tissues by quickly differentiating into both neuronal and glial cells endowed with functional competence [34, 35]. However, this postulated mechanism may not necessarily represent the true principal mechanism underlying the therapeutic efficacy of MSCs, as these cells possess limited ability to differentiate into functional neuronal cells [36, 37]. At present, the mechanisms underlying stem cell treatments are widely acknowledged to be very intricate and not yet comprehensively elucidated. Several preclinical investigations examining MSC-based therapy for brain repair have suggested possible mechanisms encompassing the following actions [33, 36, 38–40]:

(i) Fostering endogenous neuroregeneration: Exogenous neurogenesis refers to the process of implanted stem cells undergoing direct differentiation into neurons, while endogenous neurogenesis relates to the generation of neurons from the organism itself induced by implanted stem cells. Endogenous neurogenesis is more common than exogenous neurogenesis because detectable implanted cells are appreciably smaller than their original counterparts in preclinical models of ICH [39]. MSC transplantation has the ability to stimulate the proliferation and migration of endogenous stem cells. Brain-derived neurotrophic factor (BDNF) is regarded as a nerve growth factor secreted by MSCs. Ko et al. discovered that human umbilical cord-derived MSCs (HUMSCs), along with the BDNF they release, elicit therapeutic benefits in intraventricular hemorrhage; this mitigates neuronal loss and neurocognitive impairment, chiefly through the BDNF-TrkB-CREB signaling cascade [41]. Growth-associated glycoprotein-43 (GAP-43), a growth cone-specific protein in developing neurons, is known to perform a crucial function in neurogenesis. Additionally, GAP-43 has been recently identified as an axonal phosphoprotein that synchronizes with BDNF to augment its effects. According to findings reported by Cui et al., bone marrow-derived MSC (BMSC) implantation leads to upregulation of GAP-43 in the brain tissue surrounding the hematoma, thereby ameliorating neurological deficits and enhancing axonal regeneration in rats with ICH, mainly through the ERK1/2 and PI3K/Akt signaling cascades [42].
(ii) Promoting angiogenesis: Angiogenesis is a salient mechanism that underscores the therapeutic efficacy of MSC transplantation, since ischemia is common in the brain tissue surrounding the hematoma. Zhou et al. demonstrated that MSC implantation in a rat model of ICH elicited a significant increase in the expression of vascular endothelial growth factor (VEGF) in the perihematomal region, thereby driving angiogenesis [43].
(iii) Producing neuroprotective effects via suppression of anti-apoptotic pathways: A plethora of preclinical investigations have ascertained that MSCs exert neuroprotective effects by suppressing apoptosis, as confirmed via apoptosis detection assay or Fluoro-Jade staining, with several underlying pathway ramifications having been delineated. MSC implantation induces upregulated expression of protein molecules linked to the anti-apoptotic pathway, including phosphorylated Akt and B-cell lymphoma-2, while concomitantly downregulating the expression of pro-apoptotic proteins such as p38, p53, caspase 3, and Jun N-terminal kinase, all of which play a role in apoptotic cascades [44–46]. These observations collectively signal the intrinsic anti-apoptotic property of MSCs.
(iv) Preventing secondary injuries: Inflammatory responses can potentially instigate secondary injuries in instances of ICH. Accordingly, exerting control over the degree of inflammation is required to impede neurological deterioration in affected individuals. Some reports have indicated that MSCs subsequently regulate local inflammation by synergistically interacting with neighboring microglial cells, thereby lowering the concentrations of proinflammatory cytokines such as interleukin (IL)-1α, IL-1β, IL-2, IL-6, tumor necrosis factor α, and interferon γ; they also concurrently facilitate the increased expression of anti-inflammatory cytokines, including IL-4 and IL-10 [39, 47]. The worsening of neurological symptoms attributable to perifocal edema after ICH is another pivotal aspect of the secondary injury mechanism. According to studies conducted by our lab and Suda et al., MSC transplantation effectively mitigates brain edema by curbing the expression of Aquaporin4, a significant central nervous system water channel tied to the etiology of edema [46, 48]. In addition, the brain edema mechanism is associated with BBB permeability. These findings also indicate that MSCs may decrease the expression of inducible nitric oxide synthase, 3-nitrotyrosine, and matrix metalloproteinase-9, which are known to destabilize the BBB. Additionally, MSCs may promote the upregulation of tight junction markers, such as zonula occludens-1 and claudin-5, thereby preventing BBB disruption [39, 49, 50].

Interestingly, accumulating findings indicate that the therapeutic effects of MSCs are primarily ascribed to their paracrine properties, especially those involving exosomes [51]. As a safe cell-free therapy, the therapeutic potential of MSC-derived exosomes for ICH has been demonstrated. The therapeutic potential of exosomes derived from miR-133b-modified BMSCs has been revealed in vitro; these exosomes can attenuate neuronal apoptosis after ICH by suppressing RhoA expression and activating the ERK1/2/CREB pathway [52]. Moreover, Duan et al. discovered that exosomes derived from miR-146a-5p-enriched BMSCs can provide neuroprotection and improve functional outcomes after ICH by suppressing neuronal apoptosis and inflammation, which is facilitated by downregulation of the expression of IRAK1 and NFAT5 [53]. In addition to the anti-apoptotic and anti-inflammatory effects of exosome therapy, this therapy has been proven to significantly increase the number of newly generated endothelial cells, mature neurons, and myelin [54]. Overall, the underlying mechanism of exosome treatment in ICH primarily involves the angiogenic, anti-apoptotic, neurogenic, and anti-inflammatory effects mediated by miRNAs. Significantly, exosome-based cell-free therapy is expected to offer a broader approach for intervening in ICH.

Several trials, either ongoing or completed, have utilized MSCs for the treatment of ICH. As delineated in Table 1, the most widely adopted cell types in MSC-based therapy for ICH are HUMSCs and BMSCs due to the ease of their collection from patients. Notably, infusion of MSCs is generally performed during the later stages of ICH (spanning from several weeks to numerous years post ICH), and lifesaving measures are typically considered a higher priority in the acute stage. Another conceivable contingency is that the generation of ample quantities of autologous MSCs to achieve a clinically efficacious dose may be a protracted process, and it may therefore be arduous to administer a substantive allotment of MSCs to patients during the acute stage of ICH. Safety is a significant consideration in the application of novel treatment options. On the one hand, transplanted cells harbor the theoretical potential for malignant transformation into tumors [55]. Zhu et al. postulated that HUMSCs secrete soluble factors, such as VEGF, which play a critical role in fostering tumor advancement [56]. On the other hand, the transplantation procedure itself is inherently linked to the risk of complications [39]. For instance, the risk of new-onset ICH induced by injection needles is linked to intracerebral transplantation, whereas the risk of thrombosis and embolism complications is associated with venous or arterial transplantation. Nevertheless, to date, few serious adverse events have been ascribed to MSC transplantation in individuals affected by ICH, and the findings from the extant literature indicate that MSC-based therapy is a promising, safe, dependable, and efficacious strategy for ICH, promoting neural recovery and function [57–61]. Additionally, the combined therapy of MSC transplantation followed by hematoma clearance surgery has emerged as a highly promising therapeutic approach to treating ICH. In a comparative study involving 110 patients who received MSC transplantation and 96 controls within a cohort of individuals undergoing craniotomy, Zhu et al. revealed that the administration of autologous BMSCs following surgery is a salutary and risk-free modality for the treatment of ICH, endowing patients with better short-term therapeutic outcomes [62]. In a phase I clinical trial (NCT03371329), a compelling case report documented that the adjunctive protocol of MIS and MSC transplantation collectively conferred an enhancement in neurological function [63]. However, it is worth noting that current clinical investigations of MSC transplantation for ICH are presently restricted to early clinical trials and are therefore characterized by small sample sizes and modest quality. It is, therefore, imperative that future research endeavors center on designing and implementing larger clinical trials to further elucidate the efficacy and safety of this approach. Moreover, it is important to mention that translation could greatly benefit from the noninvasive assessment of stem cell survival, distribution, and function post-transplantation in vivo, especially in patients. The significant advancements in molecular imaging have enabled remarkable progress and have made it possible to image MSCs [64]. Wu et al. have developed a hybrid cell system for visually targeting transplantation and versatile ICH treatment, which involves fusing MSCs with platelets and loading them with lysophosphatidic acid-modified PbS quantum dots. The developed system synergizes the natural functions of MSCs and platelets to target ICH areas after systemic administration, exhibiting potent hemostasis, anti-inflammation, repair, and tissue regeneration effects. Most importantly, the treatment process can be visually monitored using near-infrared II fluorescence imaging, offering high spatiotemporal resolution [65]. In conclusion, future advancements could enhance imaging depth further, allowing real-time monitoring of the treatment process without requiring surgery.

**Table 1:** Application of MSCs-based therapy for ICH in humans

ID	Study type			Enrollment	Follow-up	Cell administration				Result (function/side-effect)	References
	Phase	Design	Allocation			Source	Dose	Route	Timing		
NA	I/II	Open label	Nonrandomized	*N* = 2	6 months	BMSCs	5–6 × 10^7^	IV	3 months–1 year	Improved/none	[57]
NA	I/II	Single-blind	Randomized	*N* = 4	2 years	HUMSCs	1–2.3 × 10^7^	IV	6 months–20 years	Improved/none	[58]
NA	I/II	Single-blind	Nonrandomized	*N* = 60	6 months	BMSCs	1.4 × 10^6^	IC	5–7 days	Improved/fever (5.3%)	[59]
NA	NA	NA	NA	*N* = 2	1 year	BMSCs	20 × 10^6^ cells	ICV	≥ 6 months	Improved/none	[111]
NA	I/II	Single-blind	Nonrandomized	*N* = 110	1 year	BMSCs	(6.9–12.8) × 10^7^	IT	3.01–6.89 days after surgery	Improved/fever (6.4%)	[62]
NA	I/II	Double-blind	Randomized	*N* = 5	5 years	BMSCs	8.5 × 10^5^/kg	IV	> 1 year	Improved/none	[60]
NCT02274428	I	Open label	Nonrandomized	*N* = 9	2 months	HUMSCs	0.5–1 × 10^7^	ICV	≤ 1 week	NA/none	[61]
NCT02283879	I	Open label	Randomized	*N* = 20	1 year	HUMSCs	2 × 10^7^	IV	3 months–5 years	Active, not recruiting	NA
NCT03371329	I	Open label	Nonrandomized	*N* = 12	1 year	BMSCs	(0.5/1/2) × 10^6^/kg	IV/ITV	≤ 3 days	Completed	NA
NCT02795052	I	Open label	Nonrandomized	*N* = 500	1 year	BMSCs	Unrevealed	IV/IN	≥ 6 months	Recruiting	NA
NCT01714167	I	Open label	Nonrandomized	*N* = 30	1 year	BMSCs	2–4 × 10^6^	IC	3 months–5 years	Recruiting	NA
NCT01389453	II	Open label	Nonrandomized	*N* = 0	1 year	HUMSCs	Unrevealed	IV	10–21 days	Withdrawn	NA
NCT04074408	II	Single-blind	Randomized	*N* = 100	1 year	HUMSCs	Unrevealed	ITC	1 day after surgery	Recruiting	NA

*IC* intracerebral, *ICV* intracerebroventricular injection, *IN* intranasal, injection, *IT* intrathecal*, ITV* intraventricular, *ITC* intracavitary, *IV* intravenous, *BMSCs* bone-marrow derived mesenchymal stem cells, *HUMSCs* human umbilical cord mesenchymal stem cells, *NA* not applicable

## In Situ Neuronal Reprogramming

In situ neuronal reprogramming is another exciting approach that involves converting nearby glial cells, particularly astrocytes, directly into induced neurons (iNs); iNs can replace lost neurons by introducing lineage-restricted transcription factors, microRNAs, and small molecules while also attenuating scar formation and reducing harmful inflammatory responses caused by certain astrocytes. Compared to stem cell transplantation, the utilization of endogenous astrocytes to be reprogrammed into newborn neurons is an economical, convenient, and more sustainable strategy. This approach eliminates the need for the establishment of costly stem cell banks and obviates time-consuming steps such as stem cell cryopreservation and recovery, as well as any potential for cell batch-to-batch inconsistencies. Furthermore, this approach does not have the issues of immune rejection or tumorigenesis that plague foreign cell transplantation protocols. Therefore, this strategy holds significant potential for restorative brain therapy [66].

Currently, iNs derived from patient cells have been employed in a wide range of disease models, such as Alzheimer’s disease, amyotrophic lateral sclerosis, Huntington’s disease, spinal muscular atrophy, Parkinson’s disease, Niemann-Pick disease type C, neuropsychiatric disorders, and stroke [67]. For stroke, research efforts have thus far primarily focused on ischemic stroke, with very limited attention given to reprogramming strategies for ICH. In a culture model of ICH, Feng et al. demonstrated that treatment with a drug combination consisting of valproic acid at 0.5 mM, RepSox at 1 µM, CHIR99021 at 3 µM, I-BET151 at 2 µM, ISX-9 at 10 µM, and forskolin at 10 µM led to astrocyte-to-neuron transdifferentiation and upregulation of numerous signaling molecules associated with transdifferentiation. In addition, the use of the drug combination in culture was shown to reduce oxidative stress, apoptosis, and the production of pro-inflammatory cytokines associated with ICH modeling [68]. Nonetheless, it should be noted that Feng et al.’s study has yet to be replicated in in vivo experiments; in vitro results do not always directly translate to in vivo applications. Additionally, there is currently a dearth of research regarding transcription factor- and microRNA-mediated reprogramming in ICH models, and clinical studies on this intervention have yet to be conducted. Therefore, a more comprehensive and extensive body of studies is required to evaluate the efficacy and potential mechanisms of in situ neuronal reprogramming for ICH.

## BCIs for Neurorehabilitation

**Fig. 3:**
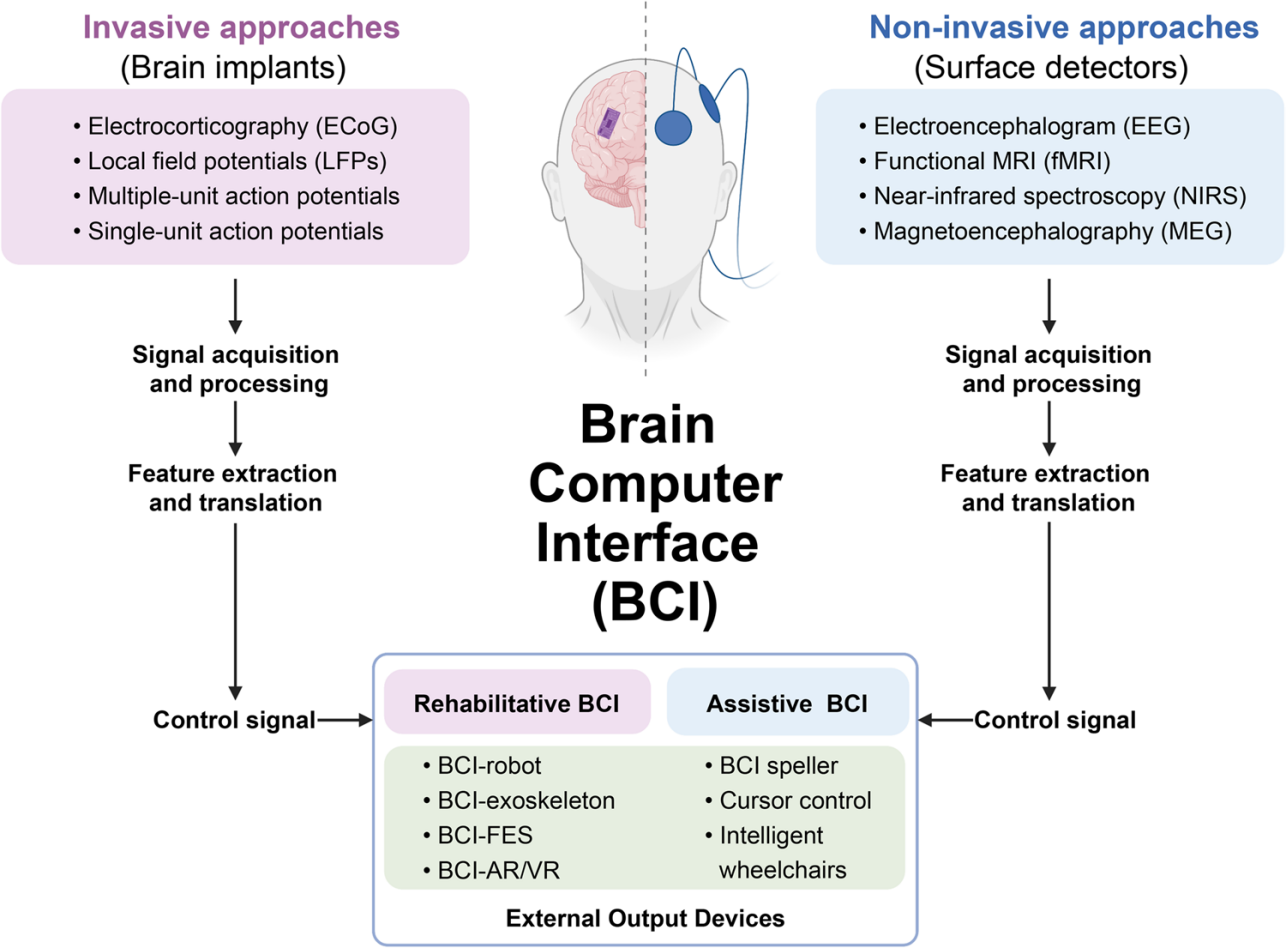
A general classification and framework for brain-computer interface systems

In the context of current-day conventional rehabilitation, ICH continues to represent one of the principal unmet rehabilitative needs. However, the integration of BCI technology into ICH neurorehabilitation represents a promising new avenue in modern rehabilitation. Compared with conventional rehabilitation methods, BCI-based neurorehabilitation strategies can dramatically enhance the ability of patients to perform tasks by decoding their intentions based on their brain activity information; BCI-based strategies allow patients to shift from passive receptivity of routine exercises to active participation in rehabilitation training and tend to produce better functional outcomes [69]. BCIs can be categorized as invasive or noninvasive, and both common types enable the detection of diverse brain signals [70]. As illustrated in Fig. 3, a typical BCI-based strategy involves several consecutive stages, including signal acquisition and processing, feature extraction and transformation, and external device output. Currently, two main classes of BCI systems have been developed to improve the quality of life of ICH patients [71]: assisted BCIs are designed to completely bypass impaired pathways, enabling patients to interact with the external world and manipulate their environment; rehabilitative BCIs are designed to restore damaged neuronal circuits and impaired neurological function by facilitating neuroplasticity. The mechanism underlying the effect of rehabilitative BCIs on neuroplasticity mainly consists of the following aspects [72, 73]: (i) neurofeedback training; (ii) reinforcement-based operant conditioning; (iii) repetitive engagement to reinforce neuronal circuits; and (iv) Hebbian learning.

## Motor Rehabilitation

Impaired motor function, mood changes, and cognitive impairment are frequently observed debilitating effects following ICH [74]. Previous research has uncovered a complex interplay between motor, cognitive, and emotional functions that may impact overall post-ICH recovery outcomes [72]. It is therefore imperative to explore a comprehensive treatment plan that addresses all of these effects; BCI technology may be well suited to achieving such an objective. Indeed, restoration of upper extremity motor impairment in patients with severe stroke was the initial impetus for the exploration of BCI techniques in the field of poststroke rehabilitation. Severely injured patients do not have the residual movement in the affected limb needed to receive the benefits of conventional rehabilitation approaches such as physiotherapy or constraint-induced movement therapy, necessitating the search for novel viable rehabilitation interventions [72]. The discovery that motor imagery can activate the same neuronal circuits as actual movement indicates that BCIs might contribute to improved neurorehabilitation [75]. Therefore, the potential benefit of the BCI-based strategy is that it only requires the patient to attempt movement. Numerous studies have shown that BCI-based interventions can improve upper extremity motor function in patients with subacute and chronic stroke [76–82]. A recent meta-analysis conducted by Yang et al. demonstrated that BCI training is an effective method for improving upper extremity motor function, with a standardized mean difference (SMD) of 0.56 [83]. Subgroup analysis further revealed that significant improvements in motor function were observed in both the subacute (SMD = 1.10) and chronic (SMD = 0.51) groups, with the subacute group exhibiting the most pronounced improvements. Notably, sensory feedback, such as haptic feedback, plays a crucial role in various aspects of motion control, particularly when performing dexterity tasks [84]. Therefore, in theory, future BCIs will require integration of information from both motor and sensory modalities to facilitate more comprehensive functional recovery of the arm and hand. It has been shown that closed-loop sensory stimulation improves BCI performance for motor recovery in a nonhuman primate model [85]. However, while motor BCI technologies have made significant strides, little progress has been made in the field of sensory rehabilitation. As such, future studies should place greater emphasis on bidirectional BCIs utilizing “closed-loop systems.” Upper extremity motor rehabilitation is critical due to its high prevalence and profound consequences limiting patients’ ability to perform daily tasks and enjoy independent social participation. By comparison, recovery from lower extremity disability using BCIs has been far less explored. Due to the scarcity of existing research studies, high-level evidence, such as that provided by meta-analyses, is not available to support this strategy, and only certain preliminary studies have explored BCI-based rehabilitation for post-ICH lower extremity disability. In the study by Chung et al. [86], 10 stroke patients (ratio of hemorrhage/ischemia: 7/3) were enrolled and randomly assigned to two groups, a control and an experimental group. In the experimental group, patients received temporally targeted functional electrical stimulation (FES) rehabilitation training when the intention of successful ankle dorsiflexion became detectable (BCI-based FES training), and patients in the control group only passively received FES (FES alone). After five consecutive days of treatment, there were significant differences in improved balance and gait function in the experimental group compared to the control group (*P* < 0.05). Similarly, Lee et al. assigned 20 patients (hemorrhage/ischemia, 11/9) during the first 6 months poststroke to either a pseudoneurofeedback or neurofeedback group, with both groups undergoing intervention for 8 weeks [87]. The results revealed significant improvements in gait parameters (velocity and cadence) in the neurofeedback group compared with the pseudoneurofeedback group (*P* < 0.05). In general, future research on motor rehabilitation should prioritize a substantial number of clinical trials and the advancement of novel systems. In addition, rehabilitation of the lower extremities should be given increased levels of attention in the future.

## Emotional and Cognitive Rehabilitation

Evidence from a large cohort study has revealed that post-ICH depression is common, affecting 23.3% of hemorrhagic patients within 2 years [88]. Depression is often overlooked in post-ICH rehabilitation even though it has been implicated in late exacerbations of ICH [89]. Post-ICH depression is typically treated by pharmacotherapy. However, post-ICH treatment often involves taking multiple medications with potential drug interactions with antidepressants. In addition, selective serotonin reuptake inhibitors, the first-line agents for moderate to severe depression, have been linked to a higher risk of bleeding and should therefore be critically considered for use in patients with ICH [89, 90]. BCIs are effective alternatives to conventional methods for the treatment of post-ICH depression [91]. There are several examples of the successful use of BCIs, demonstrating their potential for treating depression with brain stimulation. Scangos et al. designed a closed-loop invasive BCI with multichannel recording and microstimulation techniques [92]. In this study, researchers first identified personalized depression-related biomarkers and potential target sites by analyzing deep brain electrical signals. Then, capsule/ventral striatum (VC/VS) stimulation was selectively triggered when biomarkers of a pathological state were detected. Depression symptoms in each patient significantly eased over the course of the year-long treatment. Another case study developed an effective dual-target strategy for depression that stimulated the subcallosal cingulate (SCC) and the VC/VS at the same time and demonstrated that SCC stimulation lessened negative emotions and VC/VS stimulation evinced an increase in positive mood [93]. Moreover, patients with ICH often suffer from cognitive impairment [94]. Most rehabilitation methods (motor rehabilitation based on BCIs, for instance) require that the patient has a certain minimum level of cognitive function to understand and respond to the specific demands of the rehabilitation program. Thus, it is important to pay attention to post-ICH cognitive rehabilitation. BCIs have shown tremendous, albeit surprising, potential for promoting post-ICH cognitive recovery. Neurofeedback training using BCIs has shown effectiveness in mitigating cognitive decline associated with mild cognitive impairment in older adults and attention-related hyperactivity disorder [95, 96]. Unfortunately, no study has proposed a BCI system specifically for post-ICH depressive and cognitive rehabilitation. However, the aforementioned advances inspire us to contemplate the potential applications of BCI technology in neurorehabilitation for ICHs. Further investigation of these techniques in individuals with ICH, especially those experiencing emotional and cognitive impairments, is warranted.

## Prospects and Challenges

**Fig. 4:**
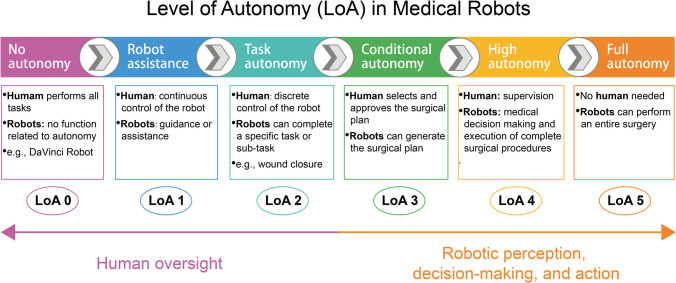
The proposed 6-stage classification of human–robot interaction and autonomy mapped to medical robots

There are six possible levels of autonomy (LoA) for medical robots, ranging from no autonomy to full autonomy (LoA 0–5) (Fig. 4) [97, 98]. At present, LoA 2 and LoA 3 are within the purview of current technology, enabling robots to undertake minor tasks under the close supervision of humans. However, for surgical robots employed in the treatment of ICH, higher levels of autonomy are needed; therefore, an intelligent decision-making model for hematoma aspiration and exceptional perceptual capabilities that can respond to multimodal information from the intracranial environment must be developed. Future endeavors should concentrate on developing a diagnosis and treatment decision-making model based on big data and artificial intelligence that integrates intracranial multimode dynamic information, such as ICP changes, detection of rebleeding and microbleeding, and real-time endoscopy images. This will ultimately facilitate personalized hematoma clearance and result in a stable reduction in ICP [99, 100].

Stem cell therapy has shown promise as a potential treatment for ICH. However, to establish the efficacy and safety of this treatment modality, more rigorous research and large-scale clinical trials are warranted. The current review has identified several limitations of stem cell therapy research that need to be addressed. First, the commonly used animal models of ICH fail to adequately capture the complex pathological processes occurring in human patients; moreover, most of model animals are healthy adults, which differ from actual ICH patients who often have other comorbid diseases. Second, obtaining autologous stem cells from patients is believed to be the most ethical and feasible source of stem cells, but the optimal route and dose of administration require further investigation. Third, the long-term safety of stem cell transplantation, especially with respect to the risk of carcinogenesis, remains a major concern that requires further investigation. Finally, it is imperative to conduct well-designed clinical trials to elucidate the optimal transplantation protocols for different stages of ICH. Although there are still uncertainties, stem cell therapy is a promising novel and effective approach for treating ICH, and ongoing research efforts are expected to overcome these limitations and ultimately lead to its clinical application.

Moreover, should in situ neuronal reprogramming indeed prove reliable, it could be adapted for neuron replacement in various disease contexts. Perhaps the most notable findings in this field of in situ neuronal reprogramming involve the nearly flawless “astrocyte-to-neuron reprogramming” achieved through the increased expression of NeuroD1 or the suppression of Ptbp1 via AAV vector or CRISPR‒CasRx [101–103]. These rapidly generated neurons exhibit exceptional potential, as their convenience, morphology, electrophysiological properties, and tissue arrangements nearly mimic those of endogenous neurons. Moreover, their ability to repair glial scars has been demonstrated. Nevertheless, there is controversy in the field of astrocyte reprogramming due to recent reports that have questioned whether newborn neurons are actually derived from transformed astrocytes. According to Wang et al., neither NeuroD1 overexpression nor Ptbp1 knockdown can reliably transform glial cells into neurons in vivo, and cells that appear to have been converted to neurons are actually preexisting endogenous neurons [104]. Similarly, Hoang et al. demonstrated that specific deletion of the Ptbp1 gene in the mouse retina and brain did not lead to glial-to-neuron transformation [105]. Yang et al. discovered that Cas13x-mediated induction of glial-to-neuron transformation after Ptbp1 knockdown was not feasible in vivo and suggested that previous “false positive” results were due to leakage of the GFAP-AAV vector [106]. In general, these findings should be validated by multiple replication studies with strict methodologies. Furthermore, some emerging technological advances suggest promising opportunities for future reprogramming research. Live imaging of an individual astrocyte’s transition into a neuron would provide incontrovertible verification of this process while simultaneously elucidating potential mechanisms within the context of brain tissue injury. Moreover, our current understanding of the transcriptional changes that occur when astrocytes convert to neurons in vivo is limited. Single-cell molecular analyses at various points during the transformation process provide valuable insights into the underlying mechanisms of this process and ultimately facilitate the determination of the degree to which iNs resemble endogenous neurons [30]. Overall, the implementation and adoption of in situ reprogramming techniques for treating various neurological disorders, including ICH, remains a challenging endeavor that requires further substantial research and practical development.

BCI-based rehabilitation has primarily been employed to address motor control impairments following ICH and has demonstrated remarkable effectiveness compared to many conventional treatment approaches, showing the potential to promote post-ICH cognitive and emotional rehabilitation. Although this novel strategy is proving to be enormously beneficial, there remain several aspects of the use of BCIs in post-ICH neurorehabilitation that require improvement. First, hybrid BCIs that use the combination of EEG and other physiological signals (myoelectricity, heart rate, etc.) as input to synergistically enhance the reliability of the detection of user intention hold promise in replacing prevailing single-signal-based BCIs [107]. Another promising future possibility is the development of asynchronous BCIs [108]. The BCI rehabilitation systems described in this review are all synchronous BCIs, which require real-time data acquisition when the user is “working” in sync with the system. In fact, in even more cases, the user exists in a “free” state. Therefore, asynchronous BCIs are introduced to identify this idle state and lead to full recovery of the autonomy of the patient. However, studies on synchronous BCIs are still in very early development, and their possible applications to clinical rehabilitation have been rarely examined. Moreover, interactions between motor, sensory, cognitive, and emotional impairments indicate the requirement for holistic BCI intervention [72]. It is believed that this increase in the rehabilitative effect produces synergistic improvements rather than a simple superposition of effects.

Nevertheless, it should be acknowledged that the aforementioned quartet of emerging technologies merely scratches the surface of what lies ahead in the realm of ICH treatment. One notable example is the potential of blood-based biomarkers, which holds promise for revolutionizing therapy breakthroughs. By enabling enhanced risk stratification and facilitating clinical decision-making for patients with ICH, these biomarkers empower the customization of standard care in alignment with the principles of personalized medicine. Lv et al. outlined blood biomarkers for ICH diagnosis, hematoma expansion, and perihematomal edema [109]. Specifically, diagnostic biomarkers play a crucial role in rapidly distinguishing between ischemic stroke and ICH before hospital admission, facilitating prompt treatment decisions [110]. Additionally, given the importance of early treatment, the identification of biomarkers related to hematoma expansion and perihematomal edema may provide essential information for initiating targeted therapeutic strategies based on personalized decisions. Indeed, the existing body of research has predominantly centered on blood biomarkers evaluated at a solitary time point, but it is crucial to acknowledge that reliance on these biomarkers alone may not fully capture the multifaceted underlying mechanisms associated with ICH. Henceforth, it is imperative for future research to gather repeated measurement data on post-ICH blood biomarkers at various time intervals to construct dynamic predictive models or decision support tools aimed at identifying the most effective management strategies for ICH.

In summary, robot-assisted MIS has provided new insights into primary brain injury following ICH, while MSC transplantation and in situ neuronal reprogramming represent novel strategies for neuroregeneration, which may also help to alleviate secondary brain injury after ICH. Moreover, BCI technology has demonstrated potential in promoting the recovery of motor, emotional, and cognitive function following ICH. The field of ICH treatment is at an exciting juncture, and future controlled clinical trials should consider contemporary techniques alongside conventional management strategies to establish a comprehensive and innovative treatment paradigm that addresses different therapeutic targets and goals at different stages of ICH.

## Supplementary Information

Below is the link to the electronic supplementary material.

Supplementary file1 (DOCX 27 KB)

## Data Availability

All relevant data are within the manuscript and its Additional files.
